# Enteritis in an Elephant

**Published:** 1884-04

**Authors:** George W. Bowler


### Art. XI.—ENTERITIS IN AN ELEPHANT.

BY GEORGE W. BOWLER, M. D. V. S.

ON THE 27th of August I was called to see the female elephant "Allah" belonging to the Menagerie of Barnum, Bailey and Hutchinson, which arrived in Cincinnati the night previous. On my arrival at the tent I was informed by Mr. George Arstingstall, the veteran trainer, that the elephant had been very uneasy during the night, lying down and getting up frequently, and had refused all food. Messrs. Barnum, Bailey and Hutchinson were quite anxious as the symptoms were precisely similar to those in the case of one of their elephants which they had recently lost in New York, the autopsy proving death to have resulted from enteritis.

On examination I found the skin, ears and extremities quite cold; tongue dry and parched; eyes sunken and dull. I will state here that the first indication of sickness in the elephant is to be found in the eye which becomes sunken and suffused with tears. The rectum was empty and very hot. An occasional thumping was to be seen on both sides of the hypochondriac region. The animal appeared very restless and to be suffering great pain, and placed herself in various postures evidently seeking relief. I at once pronounced the case enteritis, and knew from former experience with elephants that vigorous measures must be taken at once to control it or the animal would certainly die. My examination *per rectum* showed constipation and I prescribed the following formidable dose which the keeper had much difficulty in making her swallow, viz.:

℞

Adeps........................................Lb. viii.

Ol Lini........................................Cong. i.

Tr Opii........................................℥ xvi.

Sp. Eth. Nit..................................℥ xvi.

Syrup........................................O ii.

M.

The lard and oil were first melted together and the other ingredients afterwards added. The trunk was raised above her head and the mixture poured down the throat through a metal tube made for the purpose. This was accomplished without losing any of the medicine. A number of blankets were now stitched together until of sufficient length to envelop the body. These were now thoroughly saturated with hot water, wrung out, untwisted and wrapped around the body, being reheated when cold. This treatment was continued until the next morning. In the evening she became easier and drank several buckets of water, of which I allowed her all she desired. Next day she appeared to be much relieved. I now ceased the hot water applications and ordered the body to be wrapped in hot blankets. She passed a small quantity of fecal matter which was heavily coated with mucus, slightly tinged with blood and from the peristaltic action of the bowels I felt confident that we should eventually save her, as she also gave evidence of improvement by her actions toward the neighboring elephants, which she now noticed for the first time since the attack, responding to their caresses. She now ate a small piece of Jumbo bread (bread made of molasses) and some apples. A little oat-meal gruel was offered her, but she refused it. At about 10 A. M., I examined the rectum and removed several hard balls coated with mucus. This was followed in about two hours by a natural passage. The temperature became normal, the eye bright, the extremities warm and the appetite returned. It was interesting to notice the sympathy shown by her companions during her illness. In a few days she was able to take part in the performance.

